# Impact of Rescue-Thrombolysis during Cardiopulmonary Resuscitation in Patients with Pulmonary Embolism

**DOI:** 10.1371/journal.pone.0008323

**Published:** 2009-12-15

**Authors:** Fikret Er, Amir M. Nia, Natig Gassanov, Evren Caglayan, Erland Erdmann, Uta C. Hoppe

**Affiliations:** 1 Department of Internal Medicine III, University of Cologne, Cologne, Germany; 2 Center for Molecular Medicine, University of Cologne (CMMC), Cologne, Germany; Universidad Peruana Cayetano Heredia, Peru

## Abstract

**Background:**

Cardiac arrest in patients with pulmonary embolism (PE) is associated with high morbidity and mortality. Thrombolysis is expected to improve the outcome in these patients. However studies evaluating rescue-thrombolysis in patients with PE are missing, mainly due to the difficulties of clinical diagnosis of PE. We aimed to determine the success influencing factors of thrombolysis during resuscitation in patients with PE.

**Methodology/Principal Findings:**

We analyzed retrospectively the outcome of 104 consecutive patients with confirmed (n = 63) or highly suspected (n = 41) PE and monitored cardiac arrest. In all patients rtPA was administrated for thrombolysis during cardiopulmonary resuscitation. In 40 of the 104 patients (38.5%) a return of spontaneous circulation (ROSC) could be achieved successfully. Patients with ROSC received thrombolysis significantly earlier after CPR onset compared to patients without ROSC (13.6±1.2 min versus 24.6±0.8 min; *p*<0.001). 19 patients (47.5%) out of the 40 patients with initially successful resuscitation survived to hospital discharge. In patients with hospital discharge thrombolysis therapy was begun with a significantly shorter delay after cardiac arrest compared to all other patients (11.0±1.3 vs. 22.5±0.9 min; *p*<0.001).

**Conclusion:**

Rescue-thrombolysis should be considered and started in patients with PE and cardiac arrest, as soon as possible after cardiac arrest onset.

## Introduction

In Europe pulmonary embolism (PE) mortality ranges from 3.4 in Norway to 12.8 per 100.000 habitants in Austria [1]. Fulminant PE is associated with cardiac arrest in 10–20% of cases, which is the major cause of increased mortality rate of 65% to 95% [2], [3]. Cardiac arrest is driven by multiple mechanisms, most importantly by obstructive shock with severe dysfunction of the right ventricle [4], [5], [6]. PE causes up to 15% of hospital deaths [7], [8].

Thrombolysis in PE is recommended in patients with shock and hypotension [9]. However, it is unclear whether in patients with cardiac arrest and cardiopulmonary resuscitation (CPR) thrombolysis is advisable. While over years guidelines listed CPR as a contraindication for thrombolysis [10], newer guidelines recommend to consider thrombolysis therapy during CPR in patients with proven or suspected pulmonary embolism [9]. In clinical practice thrombolysis therapy is generally applied by the rescue team in the case of unsuccessful resuscitation as the “last chance” of the patient. Beside the economic concerns of this regime efficiency of thrombolysis therapy during CPR in patients with documented or highly suspected PE remains unknown. Thrombolysis in unselected patients with out-of-hospital cardiac arrest was not beneficial [11].

## Methods

### Patients

We identified patients with in-hospital witnessed cardiac arrest, who were initially admitted to the intensive or intermediate care unit of the University Hospital of Cologne with dyspnea and the clinical suspicion of PE between 2004 and 2008. Patients were included in the analysis either, when PE was verified by CT-scan, ventilation/perfusion (V/P) scintigraphy, transesophageal echocardiography or postmortem section. In case of missing objective detection of PE, patients were included when a high probability of PE was present, reflected by a Wells score >6 [12], elevated serum D-dimer concentration and additional echocardiographic signs of right heart failure. All patients received intraveneous recombinant tissue plasminogen activator (rtPA) during CPR. The time of cardiac arrest and the onset of rtPA administration were noticed in patient's charts and/or additionally in a CPR-protocol of the University Hospital of Cologne. Return of spontaneous circulation (ROSC) was defined as sufficient mean arterial blood pressure (≥65 mmHg) without mechanical thorax compressions. The neurological outcome was assessed by Cerebral Performance Categories (CPC), where CPC 1 indicates a good cerebral performance, CPC 2 moderate cerebral disability, CPC 3 severe neurological disability, CPC 4 coma or vegetative state and CPC 5 dead [13]. The Institutional Review Board of our university approved this study.

### Statistical Analysis

All variables were tested for normal distribution with the Kolmogorov-Smirnov test. Continuous variables are expressed as means ± standard error of the mean. Comparison of 2 means was performed with the *t* test for normally distributed variables and the Mann-Whitney *U* test for non-Gaussian variables. Chi-quadrat test was used for nonparametric comparisons. All statistical tests were 2-tailed, and *p*<0.05 was considered statistically significant.

## Results

### Baseline Characeristics

We identified 104 patients at age of 61.5±1.4 years with cardiac arrest and consecutive resuscitation due to verified or highly suspected PE. The baseline characteristics are summarized in table 1. In 63 patients PE was objectified by CT-scan (n = 45), transesophageal echocardiography (n = 12), V/P scintigraphy (n = 3) or postmortem section (n = 3). A mean Wells score of 7.0±0.34 revealed a high probability of PE in patients without objective verification of PE. The cardiac rhythm documented at the onset of cardiac arrest was sinus arrest or electromechanical dissociation in 61 patients and ventricular or supraventricular tachycardia or ventricular fibrillation in 43 patients. In all cases rtPA was administrated for fibrinolysis as a bolus injection in few minutes at a mean concentration of 80.5±2.4 mg. The decision for thrombolysis, dosage and time of onset was made by the rescue team.

**Table 1:** Baseline characteristics of the patients.

	All (n = 104)	Resuscitation with thrombolysis		*P*
		Successful (n = 40)	Not successful (n = 64)	
**Men (%)**	43 (41)	20 (46.5)	23 (53.5)	0.22
**Age**	61.5±1.4	64.2±2.3	60.5±1.7	0.34
**Hypertension (%)**	60 (56)	24 (40)	36 (60)	0.84
**CAD (%)**	36 (34.6)	14 (38.9)	22 (61.1)	1.00
**Congestive heart failure (%)**	37 (35.6)	10 (27)	27 (73)	0.09
**Diabetes mellitus (%)**	31 (29.8)	14 (45.2)	17 (54.8)	0.39
**PAD (%)**	14 (13.5)	6 (42.9)	8 (57.1)	0.77
**Stroke (%)**	2 (2)	0 (0)	2 (100)	0.52
**Previous PE (%)**	15 (14.4)	8 (53.3)	7 (46.7)	0.25
**Previous DVT (%)**	13 (12.5)	7 (53.8)	6 (46.2)	0.24
**PE verified**	63 (60.6)	29 (46)	34 (54)	0.06
**Wells score**	7.8±0.21	8.0±0.30	7.7±0.29	0.45

CAD indicates coronary heart disease, PAD indicates peripheral arterial occlusive disease, DVT indicates deep venous thrombosis.

### Effect of Thrombolysis during CPR

**Figure 1:**
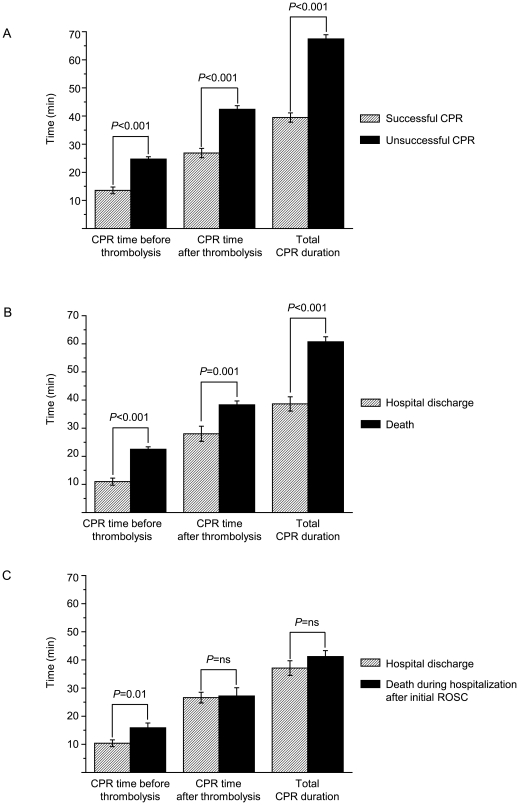
Time-outcome relation. Time of thrombolysis onset, CPR time after thrombolysis and total CPR duration in patients with and without successful CPR (A), in patients with hospital discharge vs. those who died (B), and in patients with hospital discharge versus those with secondary lethality after initially successful CPR.

In 40 (38.5%) of 104 patients thrombolysis and resuscitation was successful with ROSC. The baseline characteristics were not different in patients with and without ROSC (Table 1). The time between cardiac arrest and rtPA administration was significantly shorter in patients with ROSC (13.6±1.2 min) compared to patients without ROSC (24.7±0.8 min; *p*<0.001; Figure 1A). The mean CPR duration was longer in patients without ROSC than with ROSC (67.5±1.5 vs. 39.5±1.7 minutes; *p*<0.001; Figure 1A).

**Figure 2:**
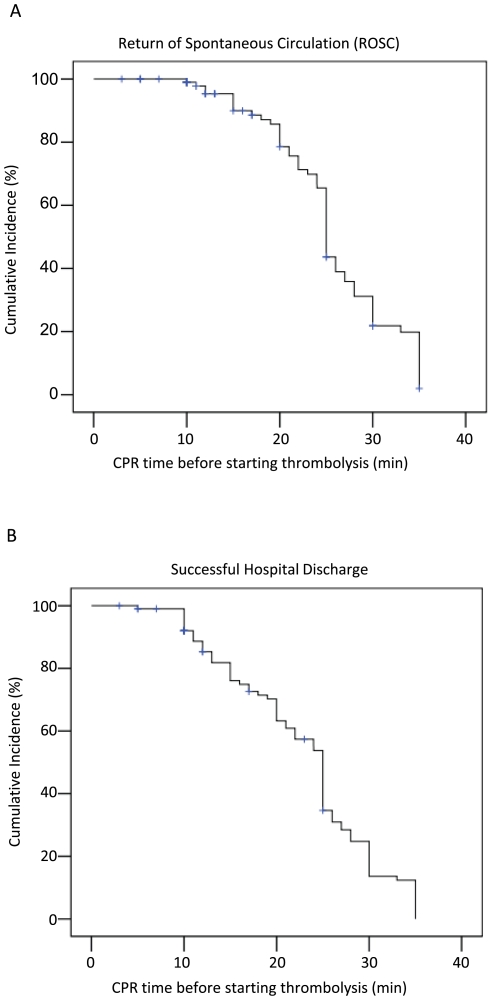
Effect of the time until thrombolysis onset on outcome. (A) Time dependent ROSC, (B) time dependent successful hospital discharge.

19/40 patients (47.5%) with ROSC survived to hospital discharge. Of multiple evaluated parameters (age, gender, comorbidities, medication, BMI, rtPA dosage) the only predictive univariate value for successful hospital discharge was the time between cardiac arrest and initiation of fibrinolysis: patients who could be discharged received thrombolysis significantly earlier compared to all other patients (11.0±1.3 min vs. 22.5±0.88 min; *p*<0.001; Figure 1B). This relation was also observed in the subgroup of 40 patients with successful ROSC, i.e. the delay of rtPA application was significantly shorter in patients who could be discharged (10.2±1.1 min) compared to patients who died later (16.0±1.7 min; *p* = 0.01; Figure 1C). Both the success rate of CPR with final ROSC and the cumulative survival to hospital discharge were higher with shorter delay of thrombolysis administration (Figure 2A+B).

### Adverse Events

Adverse events were documented only in patients who survived initial CPR (Table 2). Life threatening major bleeding with the need for blood transfusion occurred in 9 of 40 patients (22.5%). 6 of these 9 patients could be discharged. Intracranial hemorrhage was diagnosed in one patient (2.5%). A good cerebral outcome with a CPC-score <3 was present in 14 of the 19 patients (74%) who could be discharged successfully.

**Table 2:** Adverse events associated with resuscitation and thrombolysis.

Clinical outcome	Number of patients (%)
Return of spontaneous circulation (ROSC)	40/104 (38.5%)
-Intracranial hemorrhage	1/40 (2.5%)
-Blood transfusion due to major bleeding	9/40 (22.5%)
Survival to hospital discharge	19/40 (47.5%); 19/104 (18.3%)
-Cerebral Performance Categories 1+2	14/19 (73.7%); 14/104 (13.5%)

14 of 104 patients could be discharged with good neurological outcome.

### Direct rtPA Related Costs

The cost for rtPA was approximately 1200 Euro per patient. When effectiveness was measured by successful hospital discharge, a number needed to treat (NNT) of 5.5 and a treatment cost of 6000 Euro were calculated for rtPA per saved life. When assessing good neurological outcome a NNT of 7.4 and a thrombolysis cost of approximately 8.900 Euro were calculated.

## Discussion

Cardiac arrest in patients with PE is associated with a high mortality [2], [3]. Due to the persisting mechanical obstruction CPR regularly fails in these patients. Surgical embolectomy may be a meaningful approach in more stable patients but it is generally not feasible under CPR conditions [14]. Fibrinolysis seems to be a more logical alternative to resolve the obstruction. However, current guidelines do not recommend thrombolysis therapy as first line treatment option during CPR [9]. This is mainly based on the difficult clinical diagnosis of PE in the emergency settings [9], [15]. Given that reliable clinical diagnostic criteria are lacking [16], [17], a recent thrombolysis study in out-of-hospital resuscitation did not show an overall benefit in a population of patients with presumed low incidence of PE of 6% [11].

In the present investigation we minimized the risk of false diagnosis, as PE was confirmed definitely in 63 patients. Moreover in the remaining patients we used a combination of the Wells score, d-dimer values and echocardiographic right ventricular assessment to support the probability of PE.

Despite a diagnostic uncertainty in part of the patients our study provides a rather clear and simple message: when thrombolysis is considered during CPR, it should be given as early as possible. We demonstrated a highly significant association between the delay of rtPA administration and mortality.

The calculated low NNTs further suggest that the diagnosis of PE was correct in most of the 41 patients, because a low probability of PE would worsen the outcome and elevate the NNT [11]. Moreover, this observed efficacy supports the notion that early rtPA thrombolysis may be favorable in patients with cardiac arrest and confirmed PE or high probability for PE.

Thus our results justify a prospective randomized investigation with onset of thrombolysis therapy shortly after CPR initiation in this patient population.
